# Rapid, Efficient, and Universally Applicable Genetic Engineering of Intestinal Organoid with a Sequential Monolayer to Three-Dimensional Strategy

**DOI:** 10.1155/2024/2005845

**Published:** 2024-05-24

**Authors:** Weili Han, Guofang Lu, Sheng Zhao, Rui Wang, Haohao Zhang, Kun Liu, Yongzhan Nie, Jiaqiang Dong

**Affiliations:** ^1^ State Key Laboratory of Holistic Integrative Management of Gastrointestinal Cancers and National Clinical Research Center for Digestive Diseases Xijing Hospital of Digestive Diseases Fourth Military Medical University, Xi'an 710032, China; ^2^ Department of Psychiatry The First Affiliated Hospital of Xi'an Jiaotong University, 277 Yanta West Road, Xi'an 710061, China

## Abstract

Genetically modified intestinal organoids are being explored as potential surrogates of immortalized cell lines and gene-engineered animals. However, genetic manipulation of intestinal organoids is time-consuming, and the efficiency is far beyond satisfactory. To ensure the yield of the genetically modified organoids, large quantity of starting materials is required, and the procedure usually takes more than 10 days. Two major obstacles that restrict the genetic delivery efficiency are the three-dimensional culture condition and that the genetic delivery is carried out in cell suspensions. In the present study, we introduce a novel highly efficient strategy for building genetically modified intestinal organoids in which genetic delivery was performed in freshly established monolayer primary intestinal epithelial cells under two-dimensional conditions and subsequentially transformed into three-dimensional organoids. The total procedure can be finished within 10 hr while displaying much higher efficiency than the traditional methods. Furthermore, this strategy allowed for the selection of transgenic cells in monolayer conditions before establishing high-purity genetically modified intestinal organoids.

## 1. Introduction

An ideal modeling system that maintains both the *in vivo* physiological microenvironments and meanwhile allows for efficient high-throughput manipulation is critical for biological research. One such model for gastrointestinal research emerged in 2009 when Sato et al. [1] introduced the intestinal organoid culture system from Lgr5^+^ stem cells as well as intestinal crypts. Intestinal organoids are self-organizing three-dimensional structures that recapitulate both the architecture and the self-renewal kinetics of their origin tissues. The wide application of organoids derived from both normal and abnormal gastrointestinal tissues has fueled researches on stem cell biology and directed the diagnosis and management of a variety of diseases including cancer and congenital diseases associated with aberrant genetic alterations [2, 3, 4, 5].

Genetical modification of organoids allows for functional analysis of the role of specific genes in disease pathogenesis, thus making organoids promising surrogate models for the traditional immortalized cell lines or genetically engineered animals. For example, by *ex vivo* genetical manipulation, O'Rourke et al. [6] generated metastatic colorectal cancers from wild-type/mutant mouse and patient samples in a flexible, cost-efficient, and time-saving way. However, since most gene-transferring techniques were originally designed for monolayer cultures in two-dimensional conditions, efficient genetical modification of organoids embedding in three-dimensional basement membrane matrix faces huge obstacles.

So far, three different gene-transferring strategies were adopted for genetic engineering of gastrointestinal organoids, i.e., electroporation, virus infection, and liposomal mediated gene delivery [6, 7, 8, 9, 10]. All three strategies require establishment of organoids from isolated gastrointestinal crypts and subsequent preparation of dissociated single cells or cell clusters from the established organoids before gene delivery, making the procedures tedious, time-consuming, and labor-intensive. Moreover, the above-mentioned methods each hold its own inherent flaws. Electroporation relies on specific equipment that is not universally available across laboratories, and the performance of the electroporation systems varies a lot between different manufacturers according to a previous report [7]. Large quantity cells are required to ensure enough viable cells following the harsh electroporating conditions. Virus infection is efficient but usually requires high titer virus at high quantity [8, 9]. Liposomal reagent-mediated transfection is widely used and easy to perform, but the efficiency is far beyond from satisfaction [7].

Here, we present a rapid and universally applicable strategy for highly efficient delivery of genes into intestinal stem cells to form organoids. This method takes advantage of both the easy-to-handle property of primary intestinal cultures under monolayer conditions and the long-term expansion capacity of organoids in three-dimensional environment. This sequential monolayer to three-dimensional strategy shortens the preparation time for gene delivery from 1 to 2 weeks to about 4 hr while leaving out the tedious organoid manipulation procedures.

## 2. Materials and Methods

### 2.1. Materials and Animals

Six- to 8-week-old female C57BL6N mouse was purchased from Charles River and used for all experiments. All the protocols were reviewed and approved by the Fourth Military Medical University Animal Care and Use Committee (KY20183087-1) and was carried out in accordance with the National Research Council's Guide for the Care and Use of Laboratory Animals. For materials used in the study, see key resources table in the Supplementary Materials.

### 2.2. Components of Different Medium Formula

The DMEM-EI medium was as reported by Campbell. The basal medium was the Dulbecco's Modified Eagle's medium containing 0.11 g/L sodium pyruvate and 0.58 g/L glutamine, which was further supplemented with 2.5% fetal bovine serum, 0.25 IU/mL insulin, and 50 ng/mL recombinant murine EGF. For the ER, ENR, and WENR medium, the basal medium was DMEM/F12 medium containing nonessential amino acids, 150 mg/L sodium pyruvate, 125 nM N-acetyl-cysteine, 1x N2 supplement, and 1x B27 supplement. The ER formula was the basal medium supplemented with 50 ng/mL recombinant murine EGF and 500 ng/mL recombinant murine R-spondin1. The ENR formula was the ER medium supplement with 100 ng/mL recombinant murine Noggin. The WENR formula was the ENR medium supplemented with 100 ng/mL recombinant murine Wnt3a. All the formula described above contained 100 U/mL penicillin–streptomycin.

### 2.3. Intestinal Crypts Isolation

Freshly sacrificed mouse was sterilized with 75% ethanol, and about 15 cm small intestine from the duodenum was removed, cut open longitudinally, and washed in ice-cold phosphate-buffered saline (PBS) without calcium or magnesium. Before cutting the jejunum into about 1 cm pieces, the villi were scratched with glass slides. The pieces were washed with ice-cold PBS and then placed in PBS with 2 mM EDTA and incubated on an orbital shaker for 30 min at 4°C. Intestinal crypts were released by vigorously shaking the intestine pieces in PBS, and the optimal release was confirmed under microscope. The crypt suspension was sequentially filter through the 100 *μ*m and the 70 *μ*m strainers to remove large villi fragments. Finally, crypts were pelleted at 150 g for 3 min and used for establishment of primary monolayer cultures or three-dimensional organoids.

### 2.4. Primary Intestinal Monolayer Culture

The 48-well plates were precoated with growth factor reduced standard Matrigel at 1 : 40 dilution for 1 hr at 37°C to allow for the Matrigel to polymerize. About 200 crypts were seeded into each well of the 48-well plates and incubated in the abovementioned DMEM-EI, ER, ENR, and WENR medium or the commercially available IntestiCULT organoid growth medium that were supplemented with different combinations of Y-27632 (10 *μ*M), CHIR99021 (3 *μ*M), and LDN-193189 (100 nM). Four hours later, nonadherent cell fragments were washed away with the DMEM/F12 medium, and fresh growth medium was added for further culture.

### 2.5. Three-Dimensional Intestinal Organoids Culture from Crypts and Primary Intestinal Monolayers

For generation of intestinal organoids from crypts, freshly isolated crypts were mixed with Matrigel on ice at a ratio of approximately 100–200 crypts per 40 *μ*L Matrigel. Forty microliter Matrigel–crypt mix was dispensed at the center of the each well of 48-well plates which was then placed in a 37°C incubator for 15 min to solidify the Matrigel. Two hundred fifty microliters of growth medium with 10 *μ*M Y-27632 was added and replaced with the growth medium without Y-27632 every 3–4 days.

For the generation of intestinal organoids from primary intestinal monolayers, the established primary intestinal monolayer cells were dissociated into cell clusters with 500 *μ*L TrypLE Express Enzyme at 37°C for 7 min, and then 500 *μ*L DMEM containing 10% FBS was added. The cell clusters were pelleted in a 1.5 mL Eppendorf tube precoated with BSA at 4°C at 400 g for 5 min. The cell pellets were washed with DMEM/F12 twice and then resuspended in Matrigel and cultured routinely.

### 2.6. Immunofluorescence Staining

The primary intestinal monolayers or the intestinal organoids were fixed in 4% paraformaldehyde for 10 min and 30 min, respectively. The monolayers were permeabilized with 0.2% Triton X-100 in PBS for 10 min directly after fixation, while the organoids were washed stringently with PBS to remove residual Matrigel before permeabilization with 0.5% Triton X-100 in PBS for 30 min. After wash in PBS, the monolayers and organoids were block with the QuickBlock™ Blocking Buffer for Immunol Staining for 10 and 30 min, respectively. The samples were then incubated with the primary antibody at 4°C overnight. The dilution for the primary antibodies were 1 : 1,000 for villin, 1 : 500 for chromogranin A and LYZ1, and 1 : 200 for MUC2. The samples were then washed and incubated with the AlexaFluor 488-conjugated secondary goat–antirabbit antibody at room temperature for 1 hr followed by washing three times in the washing buffer. The samples were finally counter stained with DAPI and mounted with an antifading mounting buffer.

For labeling mucus-producing cells with UEA-1, the samples were directly incubated in 1 : 1,000 diluted FITC-labeled UEA-1 for 1 hr at room temperature after fixation, permeabilization, and washing. DAPI counter staining and mounting were the same as above mentioned.

### 2.7. Production and Concentration of Lentiviral Particles

HEK293T cells were used as the packaging cells which were maintained in DMEM containing 10% FBS. Plasmid transfection was performed in six well plates when the cells reached 70%–80% confluence. For each well, a total of 8 *μ*g plasmids (4 *μ*g lentiviral plasmid expressing EGFP with a CMV promoter, 3 *μ*g psPAX2 plasmid, and 1 *μ*g pMD2.G plasmid) and 10 *μ*L Lipofectamine were prepared in 250 *μ*L Opti-MEM Reduced Serum Medium separately. The plasmids and the Lipofectamine solution were then mixed together and incubated at room temperature for 15 min. The mixture was dripped onto the HEK293T cells and incubated in a humidified cell culture incubator at 37°C for 4 hr, after which the medium was replaced with normal culture medium. Forty-eight hours later, the supernatant was collected, centrifugated at 500 g for 5 min at 4°C, filtered through 0.45 *µ*m filter, and stored at 4°C. The HEK293T cells were further cultured in the normal culture medium for another 24 hr, and the second batch of supernatant was collected, processed as the first batch, and the two batches were mix together.

The lentiviral particles were concentrated with PEG-8000. Briefly, the lentiviral supernatant was mixed with the 4x PEG-8000 concentration buffer (40% PEG-8000 (w/v), 1.2M NaCl) at 3 : 1 ratio. The mixture was gently inverted every 20 min for five times and then left at 4°C overnight. The lentiviral particles were pelleted at 4,000 *g* for 20 min at 4°C. The pellets were resuspended in 400 *μ*L Opti-MEM medium, aliquoted, and stored at −80°C. For each infection of one well of 48-well plates, 20–40 *μ*L lentivirus suspension was used.

### 2.8. Lentivirus Infection

For lentivirus infection in monolayers, the cells were incubated in 48-well plates at 37°C for 4 hr, and then 250 *μ*L fresh culture medium containing 5 *μ*g/mL polybrene and 20–40 *μ*L of concentrated lentivirus particles was added to each well of 48-well plates. The cells were continuously incubated in 48-well plates at 37°C for 4 hr, after which the medium was discarded and the cells were detached to generate organoids or continued to culture as monolayer and subjected to selection with 1 *μ*g/mL puromycin.

For lentivirus infection in organoids, the organoids were incubated in 48-well plates at 37°C for 1–2 weeks before being dissociated into cell suspension, and then 250 *μ*L fresh culture medium containing 5 *μ*g/mL polybrene and 20–40 *μ*L of concentrated lentivirus particles was added to each well of 48-well plates. The cells were centrifuged for 1 hr at 4°C and followed by incubation at 37°C in a 5% CO_2_ incubator for 3 hr, after which the cells were resuspended in Matrigel matrix and seeded in 48-well plates for organoid formation at 37°C.

### 2.9. EdU Incorporation and Click Chemistry

BeyoClick™ EdU Cell Proliferation Kit with Alexa Fluor 555 was used for EdU incorporation and the click chemistry reaction according to the instruction. Briefly, EdU at a final concentration of 10 *μ*M was added to culture medium 2 hr before sample harvest. After fixation, permeabilization, and washing, the samples were incubated with the click reaction solution for 1 hr at room temperature, which was followed by washing, counter staining, and mounting.

### 2.10. Statistical Analysis

Data are presented as mean ± standard deviation (SD) as indicated. Student's unpaired *t*-test was used to determine statistical difference in quantification of control and perturbed cultures.

## 3. Results and Discussion

### 3.1. Results

#### 3.1.1. Optimization of Adult Primary Intestinal Monolayer Culture Conditions

**Figure 1:**
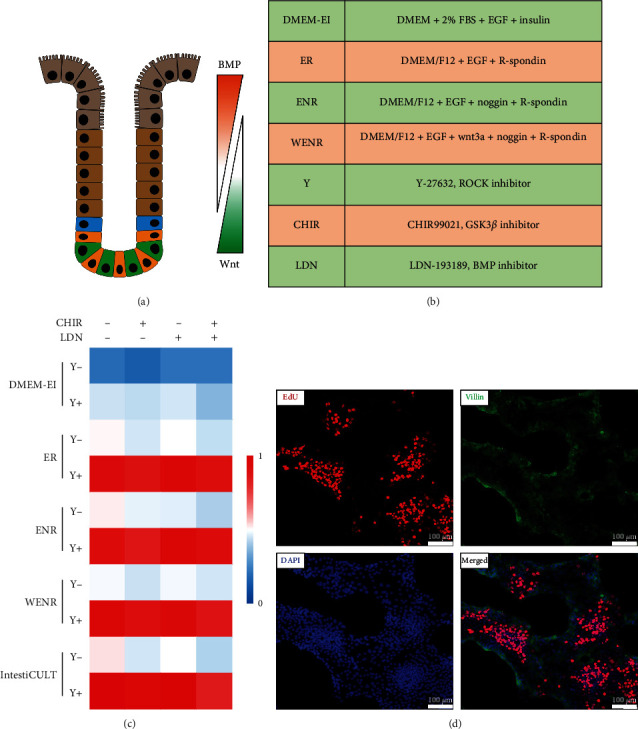
Optimization of adult primary intestinal monolayer culture conditions. (a) Schematic diagram of intestinal crypts and the opposing gradients of Wnt and BMP signaling in maintaining the homeostasis of the crypt structure and function. (b) Medium formulas and additives used for primary intestinal monolayer culture. (c) Heatmap of the normalized ratio of primary intestinal monolayer surface area of one well of the 48-well plate. (d) Representative images of the distribution of EdU- and villin-positive cells of the intestinal primary monolayer culture 24 hr after seeding.

Although the method for three-dimensional intestinal organoid culture from crypts has been well-defined, with Wnt agonists and BMP antagonists as the key components (Figure 1(a)), protocols for primary intestinal epithelial culture as monolayers vary a lot with different starting materials and medium formulas. Therefore, we started from choosing the optimal culture conditions for adult intestinal epithelial cells. Four self-prepared and one commercially available formula in combination with different signaling agonist or antagonist were tested. The DMEM-EI medium contains FBS, EGF (E), and insulin (I); the WENR, ENR, and ER medium are serum-free and are formulated from the basal DMEM-F12 medium with the addition of different combinations of Wnt3a (W), EGF, Noggin (N), and R-spondin (R) (Figure 1(b)). Among all the medium tested, except the DMEM-EI formula, there were no difference of their performance in supporting the initial formation of monolayer cell clusters as was indicated by the surface area of cell clusters. Furthermore, apart from that less and smaller monolayer cell clusters were formed shortly after plating in the DMEM-EI medium, the condition of the intestinal monolayer cells derived from adult mouse intestinal crypts in the DMEM-EI medium deteriorated quickly in the following days but continued to proliferate in other mediums tested. Supplement of the ROCK inhibitor significantly enhanced the cluster formation efficiency, resulting in more cell clusters and more cells in each cluster. Addition of the GSK3*β* and the BMP signaling pathway inhibitor either alone or in combination did not enhance or further potentiate the effect of the ROCK inhibitor (Figures 1(c) and S1). To determine whether the established primary intestinal monolayers maintain the stem cell properties, the differentiation maker villin was detected. The result suggested that most of the primary intestinal monolayer cells remain the proliferating state with only the peripheral ones expressing the differentiation marker (Figure 1(d)).

#### 3.1.2. Establishment of Three-Dimensional Intestinal Organoids from Primary Monolayer Cultures

**Figure 2:**
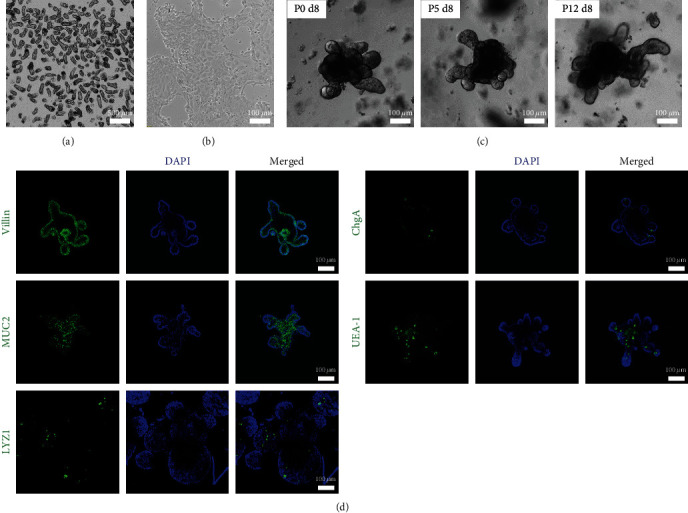
Establishment of three-dimensional intestinal organoids from primary monolayer cultures. (a) Representative image of isolated mouse small intestine crypts. (b) Representative image of primary intestinal monolayer 4 hr after seeding. (c) Representative images of organoids established from monolayers on day 8 of passage 0, 5, and 12. (d) Expression of various intestinal differentiation markers of organoids generated from primary intestinal monolayer.

To determine whether monolayer primary intestinal epithelia can be transformed into three-dimensional organoids, established monolayer intestinal cells were enzymatically detached and embedded into extracellular matrix in growth factor–rich medium with supplement of the ROCK inhibitor. Single cell or cell clusters from monolayer intestinal epithelial cultures efficiently formed three-dimensional organoids (Figures 2(a), 2(b), and 2(c)). Moreover, long-term maintenance and passages of organoids established from monolayers were achieved for at least 3 months and 12 passages (longer time was not tested, Figure 2(c)). As organoids established with the traditional method, organoids derived from monolayer intestinal cells contained all the major cell components, including the enterocytes (Villin^+^), the goblet cells (Mucin2^+^, MUC2), the enteroendocrine cells (chromogranin A^+^, ChgA), and the Paneth cells (lysozyme^+^, LYZ1) as well as mucus-producing cells (UEA-1^+^, Figure 2(d)).

#### 3.1.3. Highly Efficient Establishment of Genetically Modified Intestinal Organoid with the Monolayer to Three-Dimensional Strategy

**Figure 3:**
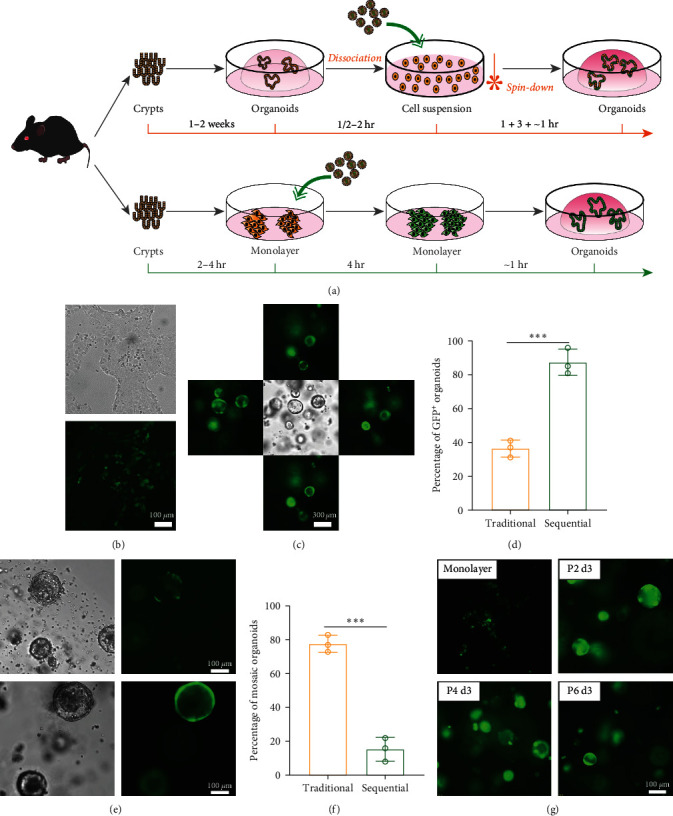
Establishment of genetically modified intestinal organoid with the monolayer to three-dimensional strategy. (a) Workflow of the traditional and the monolayer to three-dimensional strategy in the establishment of genetically modified intestinal organoids. (b) Representative image of primary intestinal monolayer cells 48 hr after lentivirus infection. (c) Representative images of intestinal organoids generated from lentivirus-infected primary intestinal monolayer cells. The images show organoids in the same field of different levels of the three-dimensional culture. (d) Quantitative analysis of the percentage of GFP-positive organoids generated from traditional and the monolayer to three-dimensional methods. The data were presented as mean ± SD,  ^*∗∗∗*^*P*  < 0.001. (e) Representative images of intestinal organoids generated from traditional (upper panel) and the monolayer to three-dimensional (lower panel) methods. (f) Quantitative analysis of the percentage of GFP-mosaic organoids generated from traditional and the monolayer to three-dimensional methods. The data were presented as mean ± SD,  ^*∗∗∗*^*P*  < 0.001. (g) Representative images of organoids derived from monolayer continuing to express GFP on day 3 in passages 2, 4, and 6.

Since electroporation equipment is not universally available across different laboratories, we tested lentivirus-mediated and liposomal mediated gene delivery. For the traditional method, three-dimensional organoids were preestablished and then dissociated into cell clusters before preforming lentivirus infection or liposomal mediated plasmid transfection. The spin-down strategy was adopted to enhance the gene delivery efficiency. For the 2D–3D sequential method, primary intestinal monolayers were prepared as mentioned above, and regular infection or transfection procedures were carried out routinely as for normal adherent cells. The traditional method takes about 1–2 weeks to complete to genetic manipulation procedures. By contrast, the 2D–3D sequential method can be accomplished within 24 hr (Figure 3(a)).

For lentivirus-mediated gene delivery, the vast majority of the primary monolayer intestinal epithelia became GFP-positive about 48–72 hr after incubation with a GFP-expressing lentivirus and successfully and efficiently formed organoids when transformed into three-dimensional cultures (Figures 3(b) and 3(c)). Nearly 90% of organoids generated from the lentivirus-infected monolayer primary intestinal cells contained GFP-positive cells, while less than half of the traditional spin-down method infected organoids became GFP-positive (0.87 ± 0.077 vs. 0.36 ± 0.050, *P*  < 0.001, Figure 3(d)). Another prominent feature is that most of the organoids derived from the monolayer culture were homogenously GFP positive, but on the contrary, a considerable proportion of the spin-down method generated organoid were mosaic ones containing both GFP-positive and GFP-negative cells (Figures 3(e) and 3(f)). In addition, the organoids derived from monolayer culture continued to express GFP for at least 4 weeks and six passages without observable reduction in the fluorescence intensity (longer time was not tested, Figure 3(g)).

For liposomal mediated gene delivery, we tested the most widely used Lipofectamine 2000 reagent and the Lipofectamine Stem reagent designed specifically for stem cells. There is no difference between the two reagents in their gene delivery capacity as evaluated by the proportion of GFP-positive cells, and both resulted in about 20% GFP-positive cells (Figure S2). Although the efficiency of liposomal mediated gene delivery is much lower than the lentivirus-mediated method, the liposomal mediated gene delivery efficiency (Figure S2) and organoid viability (Figure S2) are much higher in the 2D–3D condition than the traditional spin-down protocol.

#### 3.1.4. Enrichment of Genetically Modified Primary Intestinal Cells before Establishment of Three-Dimensional Organoids

**Figure 4:**
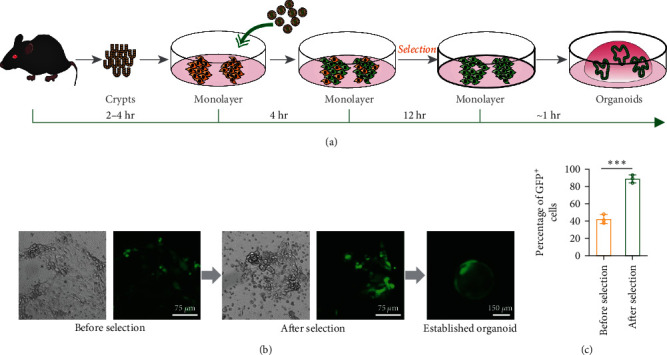
Enrichment of genetically modified primary intestinal cells before the establishment of three-dimensional organoids. (a) Workflow of the selection of lentivirus-infected intestinal primary monolayer cells and the subsequent establishment of genetically modified intestinal organoids. (b) Representative image of lentivirus-infected primary intestinal monolayer cells before and after puromycin selection and established organoid. (c) Quantitative analysis of the percentage of GFP^+^ cell before and after puromycin selection. The data were presented as mean ± SD,  ^*∗∗∗*^*P*  < 0.001.

Antibiotics or other reagent resistance-mediated selection is the most popular method to enrich genetically modified organoids. However, due to the barrier of the three-dimensional extracellular matrix, the selection process is not stable and varies a lot from batch to batch, as is indicated by the extremely wide antibiotic concentration range used in different studies. Therefore, we explore the possibility of selecting genetically modified monolayer cells before establishment of three-dimensional organoids. Puromycin successfully enriched puromycin-resistant GFP-positive primary intestinal monolayer cells, and the puromycin-enriched monolayer cells formed organoids when transformed into three-dimensional culture conditions (Figures 4(a), 4(b), and 4(c)).

## 4. Discussion

Primary intestinal epithelial culture is highly microenvironment and niche factors dependent for both two-dimensional monolayers and three-dimensional organoids [1, 11, 12]. Therefore, intestinal crypt which preserves the innate tissue structure and thus the close cellular interactions works better than single cells in establishing primary intestinal cultures [11, 12]. Among the niche factors, the opposing Wnt and BMP signaling gradient plays the fundamental role, especially the intensity of the Wnt signal [13, 14]. To meet the stringent niche factor requirement, early studies employed fetus or suckle murine intestinal tissue which holds highly intensive Wnt signals [11, 15], and in recent studies, medium formulas rich in Wnt signaling agonists and BMP signaling antagonists are usually adopted [1, 16]. However, reported formulas for monolayer primary intestinal cell culture differ a lot in the combination of Wnt agonists and BMP antagonists [15, 17, 18, 19]. Our data suggested that potentiating the Wnt signal with R-spondin alone was essential and enough to support the formation and sustained growth of intestinal monolayers from adult mouse. Nevertheless, additional factors may be needed for crypts from other segments of mouse intestine or from human intestine as additional Wnt is usually required for organoids from mouse colon as well as human small intestinal and colonic crypts.

Sufficient amount of intestinal stem cells is key for the successful establishment of genetically modified organoids. To achieve this goal, traditional methods resorted to generate and expand organoids in Wnt or GSK3*β* inhibitor containing media to boost the stemness of the cells and maintain the organoid in the cystic proliferating form instead of the budding differentiated state [7, 8, 9]. The procedure is time-consuming as it usually takes days for the crypts or intestinal stem cells to form organoids and requires to treat the organoids for at least 3 days with Wnt signal boosters before performing genetic manipulation. The procedure is also very tedious as organoids must be dissociated from the three-dimensional extracellular matrix into single cells or cell clusters. Indeed, complete removal of the extracellular matrix which interferes the genetic delivery efficiency is not easy to achieve. In the present study, we confirmed that freshly separated mouse intestinal crypts rapidly formed monolayer primary intestinal cells within hours and most of which maintained a proliferation state with only the peripheral ones underwent differentiation as late as 24 hr after plating on a very thin layer Matrigel precoated plate. More importantly, the monolayer primary cells efficiently formed organoids and maintained the long-term expansion property and the cellular diversity characteristics as those generated from traditional methods. This makes it possible to simplify the genetic manipulation procedure from operator-unfriendly three-dimensional operations to routine cell culture handling processes.

It is well-known that delivering genes into primary cells, especially primary stem cells, of various origins is much more difficult than immortalized cell lines. In traditional protocols for gene delivery into intestinal organoids, the recipient cells are dissociated ones from intact organoids, making the gene delivery process similar to that of suspended cells [7, 8, 9]. Nevertheless, the efficiency of genetic delivery in suspend cells was much lower than that of adherent cells. To overcome these shortcomings and enhance the yield of target gene positive stem cells, starting materials were usually increased, including the number of cells as well as amount of plasmid or the titer of virus. According to previous reports, organoids from four to six wells were combined to get enough cells, and half the amount of virus produced in a 15 cm dishes or a 162 cm^2^ flask was used for virus-based gene delivery [8, 9]. In the present study, we successfully turned the gene manipulation procedures back into the adherent cell condition, saving the complex and time-consuming spinoculation steps. The conversion of the gene delivery conditions from suspend to adherent state significantly reduced the number of starting cells and the amount of virus. Indeed, cells from one well of the 48 well plate and about one-tenth of the amount of lentivirus produced in one well of six-well plate were enough to generate sufficient and high-purity target gene-positive organoids.

In conclusion, performing gene delivery into freshly established monolayer primary intestinal cells and subsequentially establishing the three-dimensional organoids was a feasible and highly efficient strategy for generating genetically modified intestinal organoids.

## 5. Conclusions

The current study presented a novel strategy for gene delivery into intestinal organoids starting from freshly established monolayer primary intestinal epithelia. Much higher gene delivery efficiency was achieved compared with previously reported methods in terms of the time it takes and the proportion of target gene positive cells.

## Data Availability

To access the data underlying the findings of the study, please contact the corresponding author.

## Supplementary Materials

Supplementary Materials Key Resources Table. Supplementary methods. Figure S1: representative images of primary monolayer intestinal cells in different medium formulas and additives. Figure S2: the efficiency of liposomal-mediated gene delivery into primary monolayer intestinal cells.
