# Supplementary material for: Rapid, Efficient, and Universally Applicable Genetic Engineering of Intestinal Organoid with a Sequential Monolayer to Three-Dimensional Strategy
Source: Stem Cells Int. 2024 May 24;2024:2005845. doi: 10.1155/2024/2005845 (PMC11178405; doi:10.1155/2024/2005845)
Supplement: Supplementary Materials — Key Resources Table. Supplementary methods. Figure S1: representative images of primary monolayer intestinal cells in different medium formulas and additives. Figure S2: the efficiency of liposomal-mediated gene delivery into primary monolayer intestinal cells. [file 2005845.f1.pdf]

Supplementary Materials

Key Resources Table

REAGENT or RESOURCE	SOURCE	IDENTIFIER
**Antibodies**		
Rabbit polyclonal anti-villin	proteintech	Cat#16488-1-AP; RRID:AB_2215975
Rabbit polyclonal anti-chromogranin A	proteintech	Cat#10529-1-AP; RRID:AB_2081122
Rabbit polyclonal anti-MUC2	GeneTex	Cat#GTX100664; RRID:AB_1950958
Rabbit polyclonal anti-Lysozyme	Abcam	Cat#ab108508 RRID:AB_10861277
AlexaFluor 488 conjugated secondary goat-anti-rabbit antibody	Jackson ImmunoResearch	Cat# JAC-111-545-003; RRID:AB_2338046
**Chemicals and Recombinant Proteins**		
TrypLE Express Enzyme	ThermoFisher	Cat#12605-010
PBS	Life Technologies Gibco	Cat# C10010500BT
Penicillin-Streptomycin	Life Technologies Gibco	Cat# 15140122
EDTA 0.5M, pH 8.0	Beyotime	Cat#ST066
Advanced DMEM/F12	ThermoFisher	Cat#12634010
FBS	Biological Industries	Cat# 04-001-1A
Recombinant Murine EGF	PeproTech	Cat# 315-09
Recombinant Murine Noggin	PeproTech	Cat# 250-38
Recombinant Murine R-Spondin-1	PeproTech	Cat# 315-32
Recombinant Murine Wnt-3a	PeproTech	Cat# 315-20
IntestiCult™ Organoid Growth Medium (Mouse)	STEMCELL Technologies	Cat# 06005
Y-27632	GlpBio	Cat#GC15712

CHIR99021	GlpBio	Cat# GC16702
LDN-193189	GlpBio	Cat#GC16580
Matrigel Matrix	Life Sciences	Cat#354230
N-2 Supplement	ThermoFisher	Cat#17502-048
B-27 Supplement	ThermoFisher	Cat#17504-044
HEPES 1M, pH7.3	Beyotime	Cat#C0215
GlutaMAX	ThermoFisher	Cat#35050-061
N-acetyl-cysteine	Sigma-Aldrich	Cat# A9165
Triton-X-100	Beyotime	Cat#ST795
Bovine serum albumin	Beyotime	Cat#ST025
Paraformaldehyde	Beyotime	Cat#P0099
DAPI	Beyotime	Cat#C1005
BeyoClick™ EdU-555 kit	Beyotime	Cat# C0075
Ulex Europaeus (Gorse) Agglutinin I (UEA I), fluorescein (FITC)	ThermoFisher	Cat#L32476
Lipofectamine 2000	Invitrogen	Cat# 11668019
Lipofectamine Stem	Invitrogen	Cat#STEM00015
Opti MEM	Life Technologies Gibco	Cat# 31985070
DMEM	Life Technologies Gibco	Cat# C11995500BT
PEG-8000	Solarbio	Cat# P8260
Triton X-100	Beyotime	Cat# P0096
**Experimental Models: Organisms/Strains**		
Mouse: C57BL/6N	Charles River	
Cell line: HEK293T	ATCC	Cat# CRL-3216

**Software**		
Graphpad Prism 9	GraphPad	https://www.graphpad.com/scientificsoftware/prism/; RRID:SCR_002798
cellSens	Olympics	IX83
cellSens	Olympics	FV3000
**Recombinant DNA**		
EGFP with a CMV promoter	vectorbuilder	Cat# VB010000-9298rtf
psPAX2	addgene	Cat# 12260
pMD2.G	addgene	Cat# 12262
**Others**		
Costar® 48-well Clear TC-treated Multiple Well Plates	Corning	Cat#3548
1.5 mL Eppendorf tube	Kirgen	Cat# KG2211

Supplementary methods

A step-by-step protocol for establishment of intestinal organoids with the monolayer to 3-dimensional strategy and genetical material delivery

Step 1: Crypt isolation

1.1 Sacrifice the mouse according to the institutional regulations and sterilize the mouse with 75% ethanol.

1.2 harvest about 15cm of small intestine from duodenum.

1.3 Open the small intestine longitudinally, rinse it in ice-cold Phosphate-Buffered Saline without calcium or magnesium (PBS).

1.4 Scratch the opened small intestine with a sterilized glass slide to remove the villi.

1.5 Cut the tissue into 2-5mm pieces and wash them with ice-cold PBS gently until the supernatant is clear.

1.6 Place the pieces in about 20 mL PBS containing 2mM EDTA in a 50mL tube and incubated on an orbital shaker at low speed (~ 50-100 rpm) for 30 min at 4 °C.

1.7 Remove the EDTA-containing PBS.

1.8 Released the crypt by vigorously shaking the intestine pieces in PBS for 3-5 times and confirm the optimal release under microscope.

1.9 Filter the crypts through the 100 μm and the 70 μm strainers sequentially to remove large villi fragments.

1.10 Centrifuge the crypts at 150g for 3 min.

Step 2: establishment of intestinal monolayer

2.1 While doing step 1.6, prewarm the 48-well plate at 37 °C.

2.2 Dilute Matrigel Matrix at 1:40 using the DMEM/F12 medium and add about 200-250 μL diluted Matrigel Matrix into the prewarmed 48-well plate.

2.3 Place the 48-well plate at 37°C for 1 hour to allow for the Matrigel to polymerize.

2.4 Count the crypts from step 1.10, and seed about 200 crypts into each well of the 48-well plate with the proper medium, e.g. the intestiCULT organoid growth medium containing 10μM Y-27632.

2.5 Incubate the plate in a 5% CO_2 incubator at 37 °C for 2-4 hours until the cells are attached to the plate.

2.6 Wash away the non-adherent cell fragments with the DMEM/F12 medium.

2.7 Continue the culture the cells with fresh growth medium.

Step 3: establishment of organoids from intestinal monolayer

3.1 Detach the monolayer cells with 500ul TrypLE Express Enzyme each well at 37°C for 7 minutes.

3.2 Terminate the digestion with 500 μL DMEM containing 10% FBS.

3.3 Wash and collect the cells by centrifuging at 4 °C at 400g for 5min in a 1.5 mL Eppendorf tube pre-coated with BSA for 3 times.

3.4 Count and resuspend the cells with 30 μL Matrigel Matrix each well to generate organoids in 37°C.

Step 4: genetic manipulation in intestinal monolayer

4.1 Lentivirus infection

4.1.1 Freshly established intestinal monolayers from step 2.7 are used for lentivirus infection.

4.1.2 250 μL fresh culture medium containing 5μg/mL polybrene and 20-40 μL of concentrated lentivirus particles was added to each well of 48-well plates.

4.1.3 Incubate the cells in 48-well plates at 37 °C for 4 hours.

4.1.4 Discard the medium and detach the cells to generate organoids as step 3 or continued to culture as monolayer and subjected to selection with 1 μg/mL puromycin for 12 hours before detachment and generate organoids.

4.2 Liposomal mediate transfection

4.2.1 The transfection is the same as standard protocol for Lipofectamine 2000 or Lipofectamine Stem in adherent cell lines.

4.2.2 Incubate the cells in 48-well plates at 37 °C for 4 hours.

4.2.3 Discard the medium and detach the cells to generate organoids as step 3 or continued to culture as monolayer and subjected to selection with 1 μg/mL puromycin for 12 hours before detachment and generate organoids.

Supplementary figures

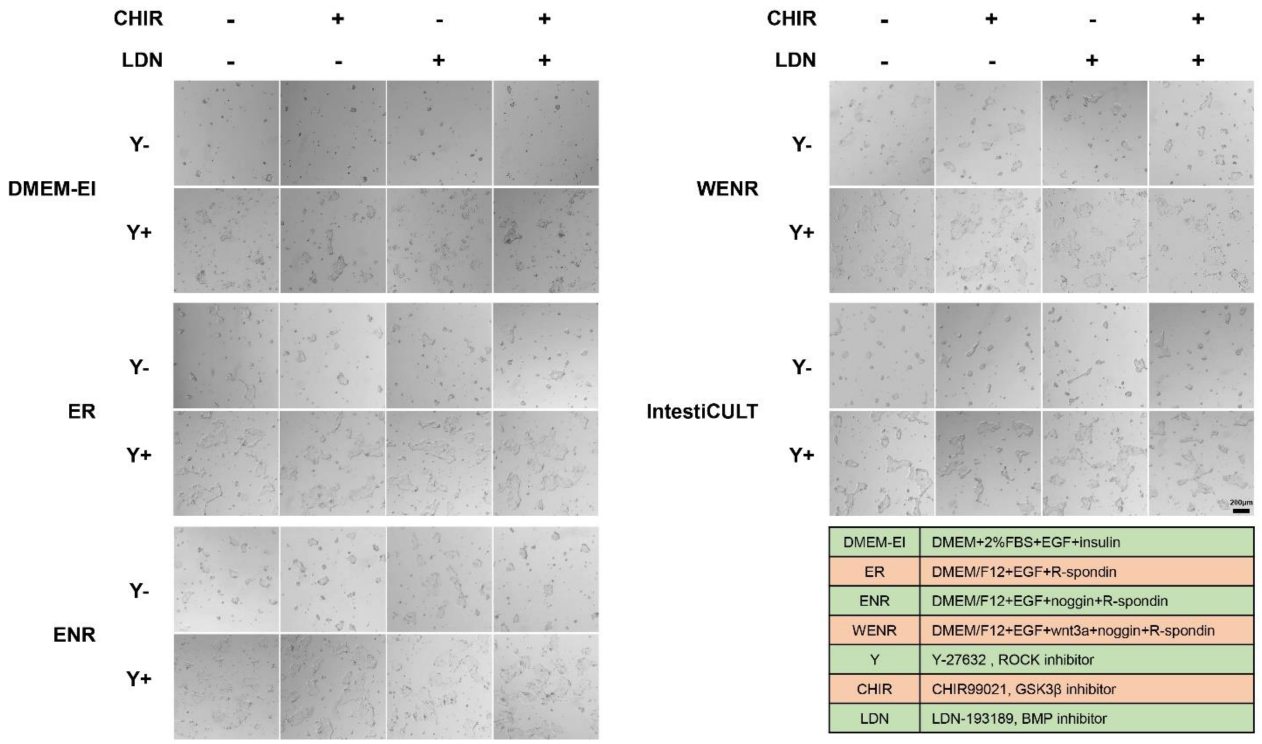

DMEM-EI	DMEM+2%FBS+EGF+insulin
ER	DMEM/F12+EGF+R-spondin
ENR	DMEM/F12+EGF+noggin+R-spondin
WENR	DMEM/F12+EGF+wnt3a+noggin+R-spondin
Y	Y-27632 , ROCK inhibitor
CHIR	CHIR99021, GSK3β inhibitor
LDN	LDN-193189, BMP inhibitor

Supplementary Figure 1. Representative images of primary monolayer intestinal cells in different medium formulas and additives.

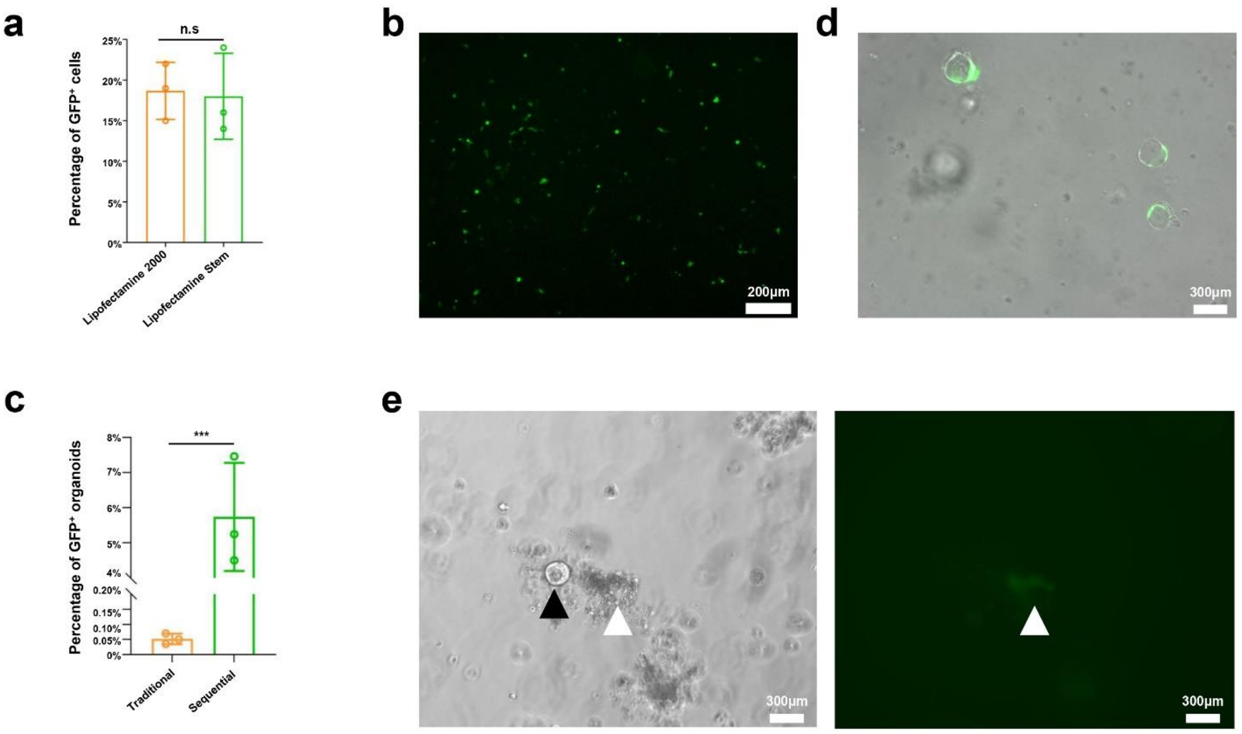

Supplementary Figure 2: The efficiency of liposomal-mediated gene delivery into primary monolayer intestinal cells. (a) quantitative comparation of the efficiency of Lipofectamine 2000 and Lipofectamine Stem reagents mediated gene delivery. The data were presented as Mean ± SD, n.s. mean not statistically significant. (b) Representative image of primary monolayer intestinal cells after liposomal mediated gene delivery. (c) quantitative analysis of the percentage of GFP-positive organoids generated from traditional and the monolayer to three-dimensional methods with liposomal mediated gene delivery. The data were presented as Mean ± SD, *** $P<0.001$. (d) representative of established organoids from primary monolayer intestinal cells following liposomal-mediated gene delivery. (e) representative of established organoids from traditional spin-down method with liposomal-mediated gene delivery, the same field of view showing an intact GFP-negative organoid (black arrow head) and a low viability GFP positive organoid (white arrow head) that under deteriorated condition.
